# Tree-insect interaction - defence response against herbivorous insects

**DOI:** 10.1186/1753-6561-5-S7-P101

**Published:** 2011-09-13

**Authors:** Hilke Schroeder, Andrea Ghirardo, Joerg-Peter Schnitzler, Matthias Fladung

**Affiliations:** 1 Johann Heinrich von Thünen Institut (vTI), Institute for Forest Genetics, 22927 Grosshansdorf, Germany; 2 Helmholtz Zentrum München, German Research Center for Environmental Health, Institute of Biochemical Plant Pathology, 85764 Neuherberg, Germany

## Background

The defoliation of oaks is an urgent problem for forestry in Central Europe. During the last outbreak of the green oak leaf roller(*Tortrix viridana*) in 2003-2005, we observed fundamental differences in the defoliation level of individual *Quercus robur* trees in Germany. Some of the trees seem to be somehow “tolerant” (T oaks) against the insects grub while some seem to be conspicuously “susceptible” (S oaks). Within this study we aim to identify the underlying molecular and biochemical mechanisms in oaks responsible for the behavioural preference of *T. viridana*. By means of combined behavioural experiments and biochemical as well as molecular analysis of preformed and induced defence mechanisms in T and S oak phenotypes, we will identify the metabolic/chemical basis of the observed differences as prerequisite for the selection of candidate genes differentially expressed in tolerant and susceptible trees, respectively, after insects feeding.

## Methods

For this purpose we until now grafted each four of the identified T and S oaks, and used them for our further experiments. The methods used at this time are as following:

1. Establishment of a “bioassay”: Larvae of the green oak leaf roller have been tested for feeding preferences on T or S oaks in a feeding choice experiment. Furthermore first olfactometer experiments with adult females were performed and the developmental performance of the larvae has been measured.

2. Preliminary analysis of emission pattern of plant volatile organic compounds (VOC) during the bioassay on T and S oaks has been done.

3. Biochemical analysis of phenolic compounds (e.g. tannins, soluble phenolic substances) and constitutive and inducible emissions of plant volatiles in T and S oaks during relevant stages of leaf ontogenesis and insect feeding and oviposition are ongoing

4. Transcriptom sequencing analysis of T and S oaks: Overall gene expression differences between the T and S oaks have been tested using biochemically defined leaf material harvested after the bioassay by next generation sequencing analysis of the complete mRNA (transcriptom). Candidate genes involved in the defence response of oaks will be identified and compared to differences in constitutive and induced patterns of phenolic compounds and VOC emissions.

## Results

We will present preliminary results of the following aspects of our study:

1. Feeding choice experiments with larvae of *Tortrix viridana*: The larvae had the choice between a leaf from T-oak and a leaf from S-oak. After 24h, the amount of feeding at each leaf was documented. We found a significant preference of the larvae for the leaves from S-oaks.

2. Olfactometer experiments with adult females: Adult paired moth had the choice between a grafted T- and S-oak in an olfactometer. After 30 minutes the decision of the moth was documented. A highly significant number of females chose the S-oaks instead of the T-oaks.

3. Performance experiment: Larvae had been fed with leaves of either S- or T-oaks only. The larvae fed with T-leaves needed much more leaf material to end up with the same weight of pupae than the larvae fed with S-leaves.

4. VOCs: In an extensive experiment the volatile substances emitted by T and S-oaks during feeding of larvae of *T. viridana* were measured online. We found clear differences in the amount of e.g. sesquiterpenes emitted by S- and T-oaks. Furthermore the S-oaks seem to emit attractants which leads to a higher amount of larvae feeding on them.

## Conclusions

We started the project with an observation, thus, we first identified phenotypes. Now, concerning the behavioural and biochemical results, we conclude that there are other factors than only environmental ones which lead to tolerant and susceptible oaks. There are physiological differences between S- and T-oaks and the identification of the candidate genes responsible for these differences is ongoing.

This approach will give us an insight into the functional genomics of *Q. robur* relating to the feeding of herbivorous insects. The question still remains unsolved whether the *Tortrix*-oak interaction is highly specific, or basics of the “*Tortrix* tolerance” can be transferred to other host-pathogen interactions. With the identification of molecular and biochemical markers of “*Tortrix* tolerance” in oaks we can contribute to decision support in sustainable forest management.

